# OncomiRdbB: a comprehensive database of microRNAs and their targets in breast cancer

**DOI:** 10.1186/1471-2105-15-15

**Published:** 2014-01-15

**Authors:** Rimpi Khurana, Vinod Kumar Verma, Abdul Rawoof, Shrish Tiwari, Rekha A Nair, Ganesh Mahidhara, Mohammed M Idris, Alan R Clarke, Lekha Dinesh Kumar

**Affiliations:** 1 Cancer Biology, Centre for Cellular & Molecular Biology, Council of scientific and Industrial Research, Hyderabad, A.P, India; 2 Bioinformatics, Centre for Cellular & Molecular Biology, Council of scientific and Industrial Research, Hyderabad, A.P, India; 3 Department of Pathology, Regional Cancer Centre, Trivandrum, Kerala, India; 4 School of Biosciences, Cardiff University, Cardiff, South Glamorgan, UK

**Keywords:** MicroRNAs, Breast cancer, Targets, 3'UTR, miRanda, TLDA

## Abstract

**Background:**

Given the estimate that 30% of our genes are controlled by microRNAs, it is essential that we understand the precise relationship between microRNAs and their targets. OncomiRs are microRNAs (miRNAs) that have been frequently shown to be deregulated in cancer. However, although several oncomiRs have been identified and characterized, there is as yet no comprehensive compilation of this data which has rendered it underutilized by cancer biologists. There is therefore an unmet need in generating bioinformatic platforms to speed the identification of novel therapeutic targets.

**Description:**

We describe here OncomiRdbB, a comprehensive database of oncomiRs mined from different existing databases for mouse and humans along with novel oncomiRs that we have validated in human breast cancer samples. The database also lists their respective predicted targets, identified using miRanda, along with their IDs, sequences, chromosome location and detailed description. This database facilitates querying by search strings including microRNA name, sequence, accession number, target genes and organisms. The microRNA networks and their hubs with respective targets at 3'UTR, 5'UTR and exons of different pathway genes were also deciphered using the 'R' algorithm.

**Conclusion:**

OncomiRdbB is a comprehensive and integrated database of oncomiRs and their targets in breast cancer with multiple query options which will help enhance both understanding of the biology of breast cancer and the development of new and innovative microRNA based diagnostic tools and targets of therapeutic significance. OncomiRdbB is freely available for download through the URL link http://tdb.ccmb.res.in/OncomiRdbB/index.htm.

## Background

Although microRNAs were discovered two decades ago, their importance as an abundant class of regulatory non-coding RNAs came to light only after their rediscovery by Reinhart *et al*., in 2000 [1]. To date, thousands of miRNAs have been identified across a range of human diseases and have been shown to be deregulated in the majority of cancers [2]. Identifying the signatures of oncomiRs in human cancer samples is extremely difficult due to the paucity of sample material. Also, cloning of these microRNAs has proven difficult due to their extremely small size and complexity of spatial and temporal expression patterns. However, the precursor transcripts of ~70-80 nt with a characteristic stem-loop fold back structure from which the miRNAs are derived, are conserved and this typical structure of precursor-microRNAs enables its identification through computational tools.

Finding the targets of a given miRNA is as important as identifying the miRNA itself to know the gene(s) it controls hence infer possible biological function through either negative or positive regulation of the target gene(s) [3]. Computational prediction of target genes for miRNAs in animals is a challenging task for both experimental and computational groups, due to the complexity in miRNA target recognition [4]. Eventhough perfect complementarity in miRNA-mRNA sequences is not required for target recognition and interaction, a small region in the 5′end (termed the seed region “position 2-8”) of miRNA is apparently needed for a near perfect base-pairing with its 3′UTR to inhibit the translation of mRNA [5].

Several databases of miRNAs, based on sequence annotation and miRNA genomics such as miRBase, [6] miRNAMap2.0 [7], miRGen [8], miRGator v2.0 [9], miRecords [10] have been developed. Amongst these databases, miRBase serves as a central database of mature miRNA sequences. MicroCosm, PhenomiR 2.0 [11] and mir2Disease [12] provide data pertinent to various diseases, whilst TargetScan [13], PicTar [14] and TargetMiner [15] are databases based on algorithms designed for microRNA target prediction for the complementary base-pairing in the seed region of the targets. TarBase [16] is a resource of experimentally validated microRNA targets, while RNAhybrid [17] is a resource based on calculation and hybridization between miRNA and mRNA. Recently, S-MED database has been created [18], which gives details of oncomiR expression profiles in sarcomas. However, there is currently no comprehensive oncomiR database for breast cancer that will provide an overview of microRNAs, their targets and the pathways that are regulated by them during breast cancer development. Our aim was to develop a database compiling all oncomiRs and their target genes involved in breast cancer and validate them using human breast cancer samples. The known oncomiRs responsible for breast cancer in two mammalian genomes, human and mouse were obtained from different databases including MicroCosm, PhenomiR and mir2Disease. The target genes were taken from the signaling pathways that are known to be affected during breast cancer. By using the miRanda algorithm [19], target genes were predicted for both human and mouse. Prediction programs including PicTar and TargetScan were used as additional sources. In nutshell, OncomiRdbB comprises of 782 human microRNAs and 246 murine microRNAs, along with 711 and 490 targets for both genomes respectively. It also provides the precise target location within the 3′UTR, 5′UTR or exon regions of the target genes. A proportion of the microRNAs have also been validated in breast cancer tissues using Taqman Low Density Arrays (TLDA). Amongst these, 34 microRNAs have not previously been reported to be associated with human breast cancer in any database. Hence, this is the first report of these novel microRNAs associated with breast cancer. Finally, using the ‘R’ algorithm and ‘GeneGo MetaCore’ (http://www.genego.com) we have characterized some of these microRNAs and their target interactions, generating examples of delineated networks and hubs of microRNA-target interaction. We envisage these network analyses will further support the identification of oncogenic targets of therapeutic value.

## Construction

### Mining of microRNAs and finding their targets

MicroRNAs of both human and mouse genomes were retrieved from different databases including miRBase, PhenomiR 2.0 and miR2Disease. A Perl subroutine was written to automate the retrieval and filtering of miRBase sequences. In order to find out the targets of these microRNAs, genes of various key signaling pathways including Wnt, JAKSTAT, *Notch, Apoptosis, VEGF* and *MAPK* were downloaded from KEGG (Kyoto Encyclopedia of Genes and Genomes) miRBase (MicroCosm), Cancer Gene Expression Data Base (CGED) [20]. The 3′UTR, 5′UTR and exon sequences of these genes were then extracted from the Ensembl database in FASTA format using the BioMart tool. miRanda *was used at 3 different energy levels (EL-15,-20 and −25) to predict putative targets for the miRNAs involved in breast cancer at the 3′UTR, 5′UTR and exonic regions of the genes of the different pathways.* The parameters used for the search were at the cutoff score of 120.

The miRanda software was initially designed to predict miRNA target genes in Drosophila. It is similar to the alignment proposed by Smith and Waterman [21], where scores were based on sequence complementarity and not on sequence identity. These targets were further confirmed using the TargetScan and PicTar algorithms.

### Experimental validation using Taqman Low Density Arrays

Taqman Low Density Array (TLDA) was performed with 50 individual human samples followed by confirmation with LNA arrays. Reverse Transcription reaction was carried out using TaqMan MicroRNA Reverse Transcription Kit (Applied Biosystems, USA) followed by PCR Reaction. Real-time PCR was performed using Applied Biosystems 7900- TLDA Real-Time PCR System. Each TaqMan Assay was run in quadruplicate. All the samples displayed good RIN value, linearity (R2 > 0.96), good abundance (average CT range 22–28) and NTC CT >38. MicroRNA profiling was done using TaqMan MicroRNA Arrays, which contains 667 human microRNAs covering Sanger miRBase version10. A pre amplification step of cDNA with preamp megaplex pool primers was performed to enhance the ability to detect miRNAs with low expression. The TaqMan human microRNA arrays consisted of 2 plates: pool A and pool B. RNU 46 and RNU 48 were used as endogenous controls and are included for data normalization. One TaqMan microRNA Assay, not related to human, was also included as a negative control. The set enabled accurate quantification of 667 human microRNAs. Reverse transcription followed by PCR reactions were carried out in ABI 7900 HT. The results were analysed using spotfire (statminer) software and the data was normalized using RNU 46 & RNU 48 as endogenous controls. Fold changes were represented as log _10_ 2 ^- Δ Δ CT^.

### Pathway analysis of miRNA-Target

Functional enrichment analysis of microRNAs and their target genes was performed to identify unique, similar and common sets of genes/proteins. Enrichment analysis of miRNAs and their targets in oncogenic signaling pathways was performed using the GeneGo MetaCore software. This software is a web-based program and performs functional analysis of experimental data or computationally compiled datasets on ontologies such as GeneGo process networks, GeneGo diseases, disease biomarkers, gene ontology processes (GO) metabolic processes, various signaling pathways, canonical pathways, protein-DNA interactions, protein-protein interactions etc. This enrichment analysis creates a degree of relevance of the datasets to the GeneGo MetaCore suite, defined by *p*-values where lower *p*-values were assigned higher priorities. The statistical enrichment analysis of miRNAs and their targets were performed based on the *p*-values generated. We have used ClustalX to identify sequence similarity with a conserved biological function of miRNAs in breast cancer.

### ‘R’ algorithm

‘R’ is an open source statistical language for statistical analysis and graphics. It provides a wide variety of statistical and graphical techniques (linear and nonlinear modeling, statistical tests, time series analysis, classification, matrix manipulation and clustering). In this study R (v2.14) packages (ggplot2) [22] were used for building the network of miRNA and their target genes. To build-up the miRNA-target interaction network we constructed a matrix using the binary concept 0 & 1. As an example, hits between microRNAs and specific target genes were assigned a binary value of 1, while 0 implies absence of recognition. This matrix was uploaded in ‘R’ which generated a graphical miRNA-target interaction network. The ‘R’ program was used to generate such types of network where a single miRNA was hitting several targets or where several miRNAs hit single target in each oncogenic signaling pathway.

## Contents

### miRNAs responsible for breast cancer

**Figure 1:**
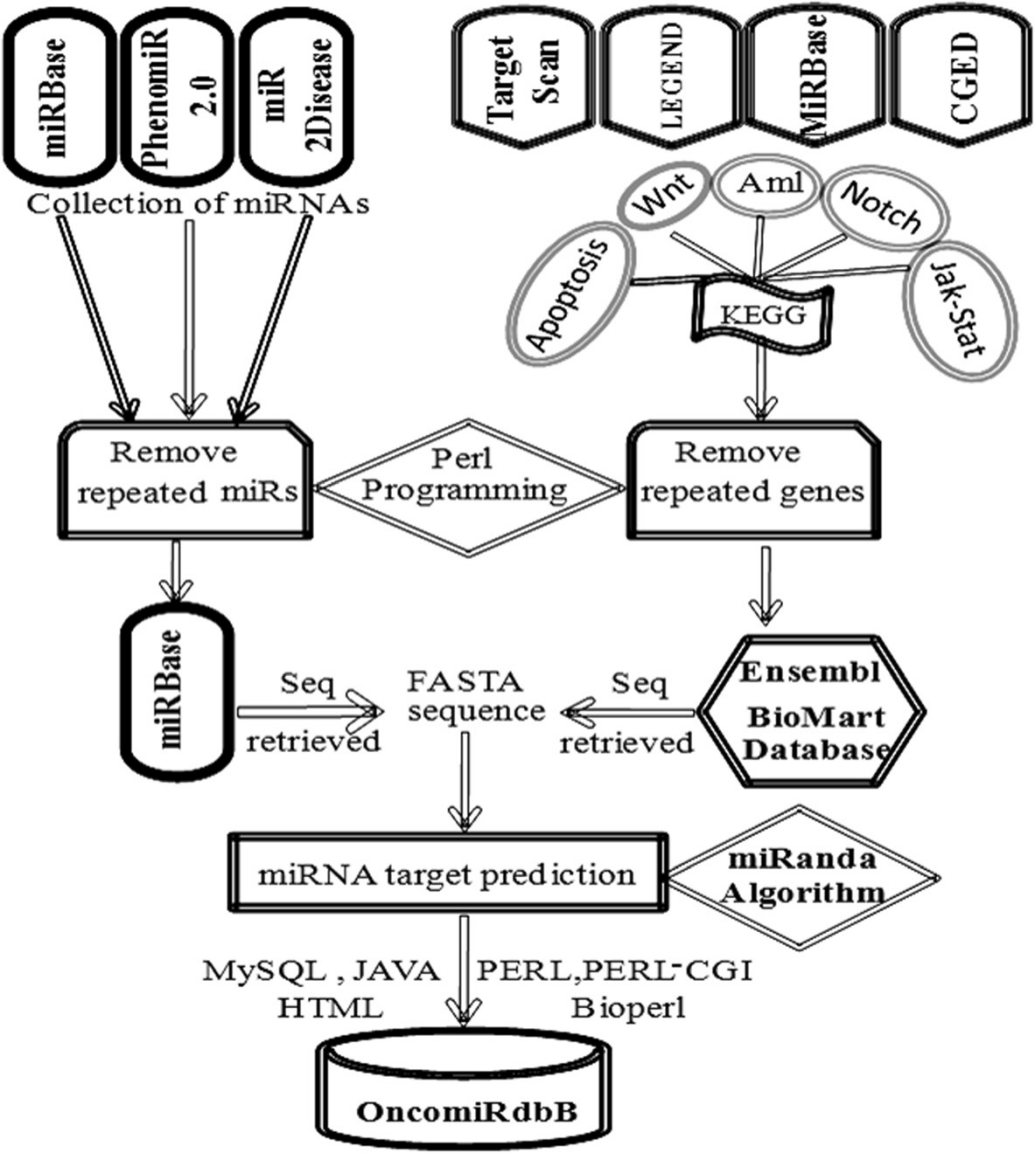
**Schematic illustration of construction of OncomirdbB using different bioinformatics approaches.** MicroRNAs were mined from different databases like miRbase, PhenomiR2.0 and miR2Disease. In order to find targets for these miRNAs, different pathway genes were downloaded from KEGG database and removed the repeated entries by using Perl script. We used miRanda for finding the targets at different energy levels.

**Figure 2:**
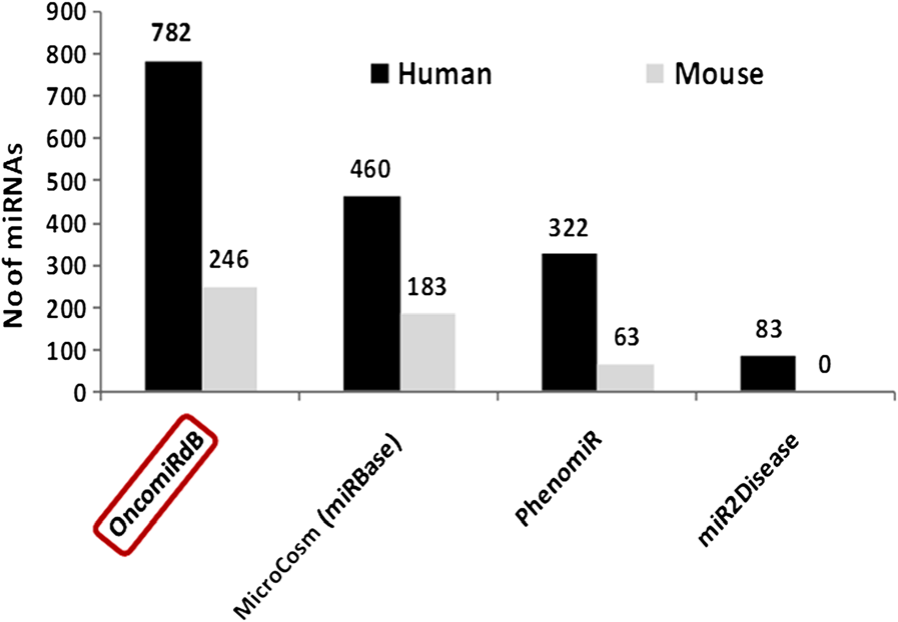
**Comparison of human and mouse miRNAs from different databases with oncomiRdbB.** Human and mouse miRNAs were mined from various databases listed and compiled as OncomiRdbB. This database has a maximum number of miRNAs compared to MicroCosm, PhenomiR and miR2Disease which list 460, 322 and 83 human miRNAs and 183, 63 and 0 mouse microRNAs respectively.

The mined miRNAs were classified as breast cancer miRNAs based on their complementarity to the target genes of different oncogenic pathways (MicroCosm) deregulated in breast cancer. This was further confirmed using miRanda, TargetScan and PicTar (Figure 1). A comparison between OncomiRdbB and existing databases demonstrated the utility of integrating various databases into one comprehensive database (Figure 2). A phylogenetic functional relationship between microRNAs of human and mouse was established by aligning the sequences using ClustalX. A conserved biological function of breast cancer microRNAs between mice and human genomes was observed after clustering both data sets. In the cladogram, branches from the same node represent descendents of a similar ancestor or cluster of the same family indicating their origin from a common ancestor. For example, hsa-miR-145, miR-151-3p and miR-30 families align with their mmu-miR counterparts indicating a conserved biological function in breast cancer development in both the genomes (Additional file 1: Figure S1). We retrieved a total of 782 human and 246 mouse microRNAs and their respective sequences associated with breast cancer from existing miRNA databases including miRBase, miR2Disease and PhenomiR. We have validated these miRNAs with two different platforms, Taqman low density arrays consisting of 667 human microRNAs (version 10) and LNA arrays using human breast cancer samples of grade 2 and grade 3 each consisting of Stages I to III. Approximately 400 significant and valid miRNAs lighted up in one or the other grades/stages classifying them as breast cancer microRNAs.

### Identification of novel microRNAs using Taqman Low Density Arrays and LNA microarray

**Figure 3:**
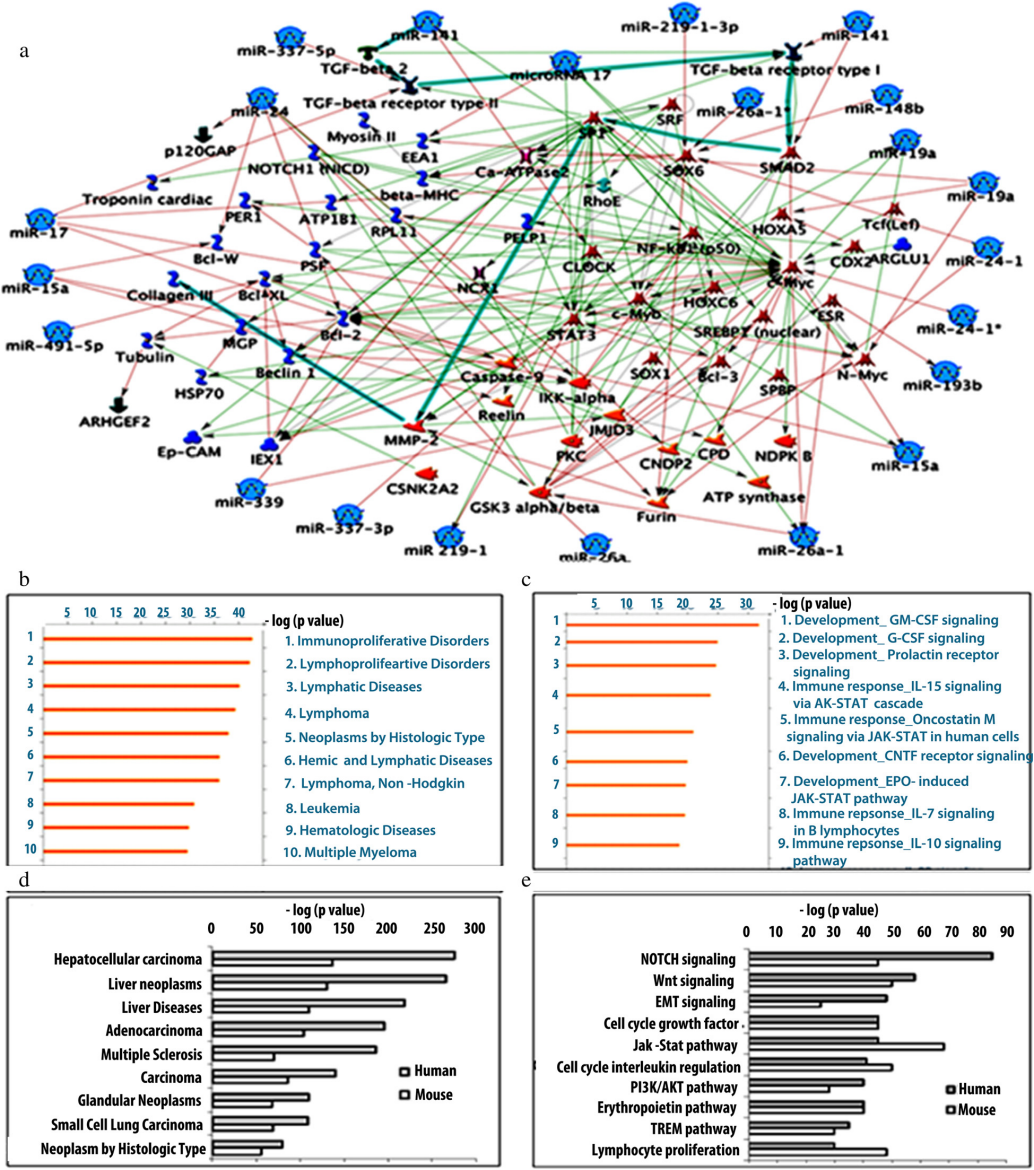
**Interaction and enrichment analysis of the novel miRNA. a**. The interaction of novel miRNAs with various signaling molecules reveals positive and negative regulation with downstream effector molecules as shown by MetaCore analysis. Red arrow indicates a down regulation whereas green arrow indicates up regulation of a particular pathway by the given miRNA ( generic binding protein;  microRNA; - Transcription factor; - receptor ligand; - generic enzyme; - protein and - regulators). **b**. Histogram representing enrichment analysis of these novel miRNAs in different diseased conditions in humans. **c**. The spectrum of novel miRNAs involved in GM-CSF signaling. **d & e**. Enrichment analysis of miRNAs and their targets shows the list of different signaling networks they are involved and are deregulated in different carcinomas.

Among the 400 significant miRNAs validated experimentally using 2 array platforms, a set of 34 novel miRNAs was identified as novel breast cancer miRNAs since they were not classified as breast cancer miRNAs earlier. The targets of these miRNAs were also identified using miRanda at 3 energy levels, EL-15, -20 and −25 and the most stringent ones (EL −25) are listed in Table 1. These miRNAs and their targets were implicated in various signaling pathways, as revealed by the MetaCore software (Figure 3a and Table 1). The network of interactions showed involvement of key oncogenic signaling pathways like Wnt, JAK-STAT, PI3K and AKT. A spectrum of miRNAs including miR-337-5p, miR-17-1, miR-15a, miR-491-5p, miR-339, miR-337-3p, miR-241, miR-19a were predicted to down regulate oncogenic targets like TGFβ, BCLXW, BCL-Xl, STATs, c-MYC and SMAD (as represented by red lines). It was also observed that miR-141 positively controls the canonical pathway involving SP1 transcription factor via TGFβ, receptor, which activates matrix metalloproteinases, the deregulation of which plays a crucial role in metastasis.

**Table 1:** Lists the experimentally validated novel microRNAs and their putative targets in different signaling pathways at EL −25 ΔG kcal/mol

**a.**				
**Novel miRNA**	**Targets(Jak-Stat)**			
hsa-miR-193b-5p	SOCS-2, AKAP13, AKT2, BCL2L1, CLCF1, IL10RA, IL10RB, JAK3, LIF, PIAS4,PIK3R2, PIK3R5, PRLR, PTPN11, SOCS7, STAT5B, TSLP, TYK2			
hsa-miR-296-3p	AKT2, BCL2L1, CSF3, GRB2, IFNGR2, IL10RA, IL11, IL11RA, IL9R, PIK3CB, PIK3R2, PIK3R5,PRKAR2A, PTPN6, STAT5A, STAT6, TSLP, TYK2			
hsa-miR-296-5p	CREBBP, GRB2, PIAS2, PTPN11, SOCS3, STAT6			
hsa-miR-339-3p	IL20RA, SOCS2, STAT5A, CLCF1, GH2, GHR, IL2RB, IL4R, IL6R, LIF			
hsa-miR-491-5p	CBLB, CISH, CSF3R, PIK3R2, PTPN6, SOCS7, SPRY4, TYK2, AKT1, IKBKG, PIK3R5, SERF2, AKAP13, CSF3, IFNAR2, IL15RA, IL2RB, IRF9, LEP, PIK3R5, PRLR, SOCS2, SPRY3, STAT6, STS			
hsa-miR-623	AKAP13, AKT2, BCAS1, CSF3, CSF3R, GRB2, IFNAR2, IL10RA, IL11RA, IL28RA, IL4R, IL6ST, PIK3R2, PIK3R5, SASH1, SOCS3, SPRY4, STAT5A, STAT6, TYK2			
hsa-miR-639	CCND1, IL2RB, PIAS4, PRLR, AKAP13, BCL2L1, CBL, CISH, CNTFR, CSF2RB, CSF3, GRB2			
hsa-miR-770-5p	IL4R, PIK3R3, AKAP13, AKT2, CBL, CNTFR, CSF3, IL11RA, IL6ST, IL7R, PIK3R5, PTPN11, SOCS2, STAT5A, STAT5B, STAT6, STS, TYK2			
hsa-miR-941	CSF2RA, CTF1, IFNAR1, IFNAR2, IL10RA, IL10RB, IL11RA, IL28RA, LIF, OSM, PIK3CA, PIK3R2, PRKAR2A, PRLR, STAT5A, SOCS3, SPRED2			
hsa-miR-15a-3p	BCAS1, FZD4, PIK3R1, AKAP13, BCAS1, CISH, CSF2, CSF3R, GRB2, IL13, IL13RA1, STAT5A, TYK2			
hsa-miR-141-5p	AKT2, BCL2, PRKAR1B, TRAF2, XIAP, CISH, IL22RA2, PIK3R3, PIM1, STAT2, STAT6			
hsa-miR-148b-5p	IFNAR2, JAK1, DFFA, IFNAR2, JAK1			
hsa-miR-17-3p	EXOG, IRAK3, TRAF2, CBL, STAT1 IL15RA, IL19, STAT6			
hsa-miR-188-3p	AKT1, OSMR, SPRY2, SPRY4, CCND1,IL10RA, CBLB, CISH, CNTFR, CSF2RB, IL13, IL13RA1, IL6, IL9R, PTPN6, SPRY3, STAT3, TYK2			
hsa-miR-218-2-3p	AKAP13, AKT2, PIM1, STAT5A, TYK2, CSF2RB, CSF3R			
hsa-miR-219-1-3p	EXOG, AKAP13, AKAP13, IL7R, PIM1, SPRY3, TSLP, IL11, IL13RA1, MATR3, IL19			
hsa-miR-24-1-5p	PIK3R1, IL19, CSF2RB, DFFB, PIK3R1, NRG1			
hsa-miR-329	SOCS2, IL10RA, IL4R, GRB2, IFNAR2, IL11RA , JAK3			
hsa-miR-337-5p	AKT2, CBL, IL10RB, TNFRSF10B, AKAP13, AKT1, CSF2RB, IL10RA, IL13RA1, PIK3R5, STAT6, CTF1, IL28RA, JAK3, STAT5A, STAT5B, STS, TYK2 , MVP			
hsa-miR-506-3p	PIAS2, STAT5A, CCND2			
hsa-miR-519e-3p	AKT2, AKT1, STAT3, STAT5A, IKBKG, NF2			
hsa-miR-521	AKT1, PIK3R1, STAT5A, CBL, CCND2			
hsa-miR-550a-3p	AKT1, CSF3, JAK3, STAT3, CASP7, DFFA, DFFB, EXOG, IKBKB, IRAK4, PPP3R1, PRKAR2A, IL10RA, IL28RA, IL2RB, PRKAR2A, IL6R			
hsa-miR-617	IL11, IL19, IL4R, LIF, PRLR, SOCS7, IL15, IL6R, AKT2, IL21R, STAM2, STAT3, IKBKG, XIAP			
hsa-miR-644a	AKAP13			
hsa-miR-934	CSF3, IL11RA, LIF, PTPN11, SOCS2, PIK3R2, AKT3, OSM, TYK2, APAF1, CASP7, IL1R1, SERF2, TRAF2			
hsa-miR-147b	XIAP, PIK3CG, STAT5B, CCND1, CCND3, CISH, CLIC6, IL19, JAK1, SPRED1, TYK2			
hsa-miR-33a-3p	IL15, EXOG			
hsa-miR-146b-5p	AKT1			
hsa-miR-19a-5p	CBL			
hsa-miR-26a-1-3p	AKT2, TYK2, PIAS3			
hsa-miR-493-5p	AKAP13, KIT			
hsa-miR-562	IKBKB, ADAM17, NMI, TGFB1, TNFSF13B, PTK2, AKT2, CSF3, KIT			
**b.**				
**Novel miRNA**	**Targets (MAPK)**	**Targets(NOTCH)**	**Targets (VEGF)**	**Targets (Wnt)**
hsa-miR-296-3p	F2RL1, NFATC2, EPHB4	EPHB4	F2RL1, NFATC2	NFATC2, VANGL1
hsa-miR-296-5p	APC, CSPG4	-	AQP1	ZEB2, APC
hsa-miR-339-3p	WEE1, FLNA	-	-	CACYBP
hsa-miR-491-5p	PDGFRA, XIAP, KAT6B	-	LRP6, PDGFRA	XIAP, LRP6
hsa-miR-770-5p	-	MAGEA1	RBM39	-
hsa-miR-941	-	-	STAT5A	-
hsa-miR-15a-3p	ARF1, WNT5A	-	RBL2	SETD8, WNT5A, VIM
hsa-miR-141-5p	FLOT1	-	IL17F	SULF1
has-miR-148b-5p	LRP1	-	LRP1, KCNH1, DNMT1	LRP1, PLCB2
has-miR-17-3p	CXADR, DUSP22, PLK1	GDPD5	PLK1	-
has-miR-188-3p	YWHAZ	MUC16, GLI1	GLI1, PIK3R2, PIK3R2	GLI1
has-miR-218-2-3p	FGF4, TNC	-	TNC	CSNK1E, PLCB2
has-miR-219-1-3p	NOS2	-	NOS2	CHST11
has-miR-24-1-5p	NRG1	-	NRG1	-
has-miR-329	CDH13	JAK3	JAK3	-
has-miR-337-5p	RARA, MVP, DUSP22, VEGFA, ABCA1, GRIN1, MAPK12, F10, BCL6, IGFBP5	CDKN1A, NCSTN, VEGFA, WNT3A	VEGFA, MAZ, LIMK1, MAPK12, F10, COL1A1, PIK3R1, IL15, IGFBP5	AXIN2, MBD2, CDH11, WNT3A, BCL6
has-miR-506-3p	-	-	-	CCND2
has-miR-519e-3p	NFKB2, RARA, NF2, TFF2, ARRB2	ARRB2	-	ARRB2, NF2
has-miR-521	-	-	NFATC1	NFATC1
has-miR-550a-3p	DYNLL1, IL6R	LFNG	IL6R, IL15	ING4, SFRP5
has-miR-617	OCLN, LRP1, NDFIP1, TIMP1	NCOR1	OCLN, MMP2, LRP1, TIMP1, ARF6	LRP1, TIMP1
has-miR-644a	-	-	-	CUX1
has-miR-934	KCNMA1	GLI1	GLI1, APEX1	GL1
has-miR-33a-3p	FLT4	FLT4	FLT4	AXIN1
has-miR-146b-5p	NFATC2, XIAP	-	NFATC2	XIAP, NFATC2
has-miR-26a-1-3p	AGER, ERBB, ACAT1, CXCR2, PRMT1, SMAD3, APOBEC3G, RRAS, PLAUR, LRP1, PGR, ERBB3, MAPK12, TRAF4,	NOS3, NOTCH4, PLAUR, FURIN	AGER, NOS3, NOTCH4, PARG, PLAUR, ERBB3, LRP1, CD47, MAPK12, FURIN, SMAD3	LRP1, ERBB3, MCC, SCN5A, PRMT1, ZNF703, SMAD3
has-miR-493-5p	PODXL, GRIN1, KIT, TGFB3	-	MUC1, KIT	FERMT2, IGF2BP1, PLCB2
has-miR-562	CDH5, PTK2, FLNA, SP1, CSF3, TGFB1, NFATC2, CD36, ERCC1, ADAM17, ARRB1, NGFR, HSPB1, MAP3K11, PTK2, TNFSF13B, APOB, KIT, CSF3, AKT2, TRAF2, ELK1, CD274, CAMK2B	ADAM17, ARRB1, TGFB1	ADAM17, APOB, HSPB1, PTK2, CSF3, AKT2, CDH5, TGFB1, NFATC2, PPARA, VASH1, SP1, KIT	CHST11, TGFB1, NMI, FXYD5, NFATC2, CAMK2B

a. The genes targeted by these miRNAs in the Jakstat pathway. b. represents those genes targeted in Mapkinase, Notch,Vegf and Wnt signaling pathways.

### miRNA targets

**Figure 4:**
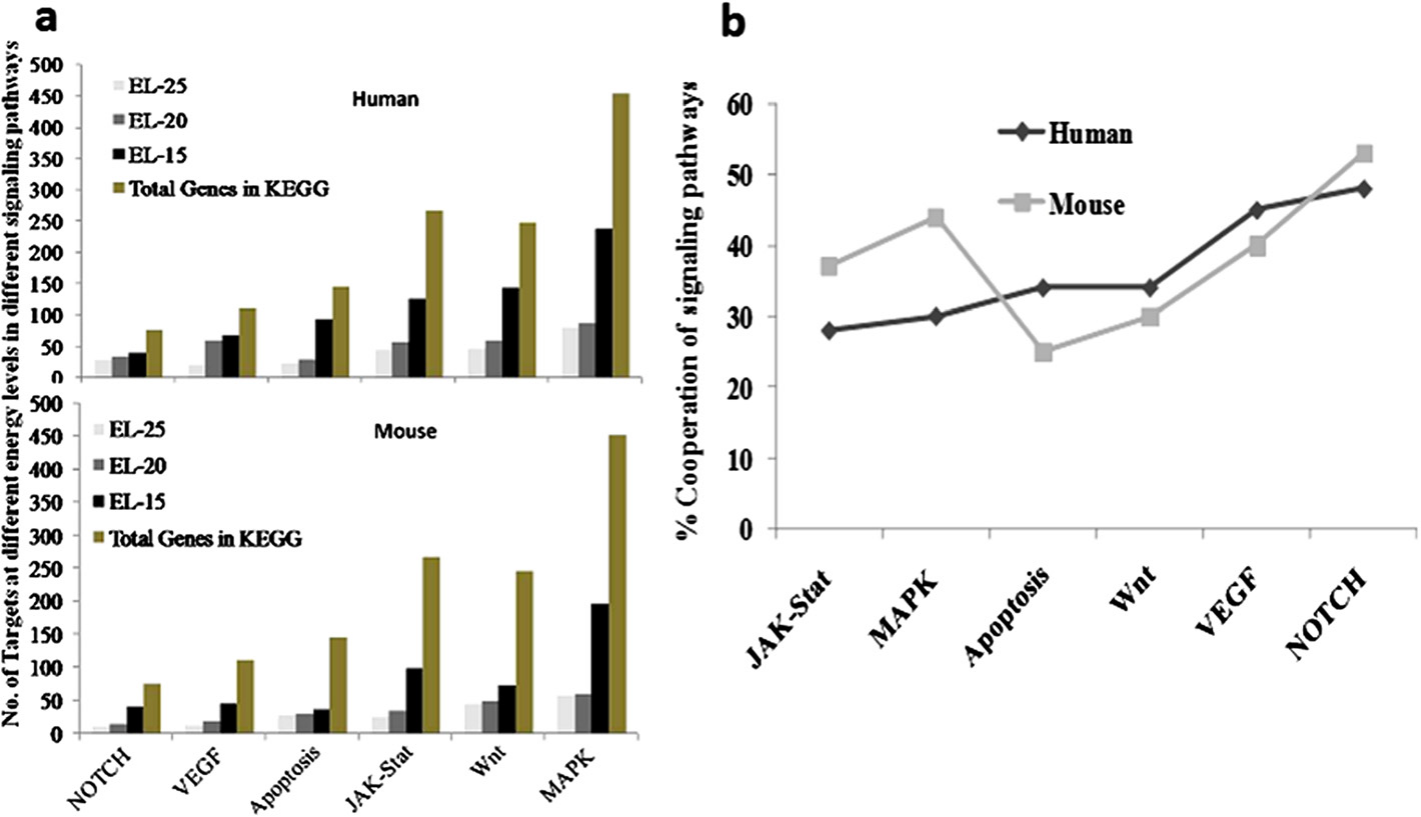
**MiRNA target identification at various energy levels in different pathways. a**. Target identification of microRNAs at 3 different energy levels was performed on the retrieved sequences from different oncogenic signaling pathways using miRanda. **b**. shows the percentage cooperation of different microRNA targets of these pathways involved in the development of breast cancer.

The miRanda program was used to predict microRNA targets in the various oncogenic signaling pathways. This algorithm was run at energy level EL-15 and 711 genes in human and 490 in mice were identified. The stringency level was increased by decreasing the energy levels to EL-20 and EL-25, in order to increase the accuracy and to identify fewer targets with increased specificity (Figure 4a). By computing the ratio of miRNA targets with the total number of genes in the respective pathways, the percentage cooperation among different pathways including the Notch, VEGF, Wnt, MAPK, Apoptotic and JAK-STAT pathways was deciphered and thus their potential involvement in breast cancer development. Notch signaling pathway was found to have the highest (~50%) percentage of cooperativity between the novel miRNAs and their signaling molecules compared to other pathways analyzed in both murine and human hosts (Figure 4b).

**Figure 5:**
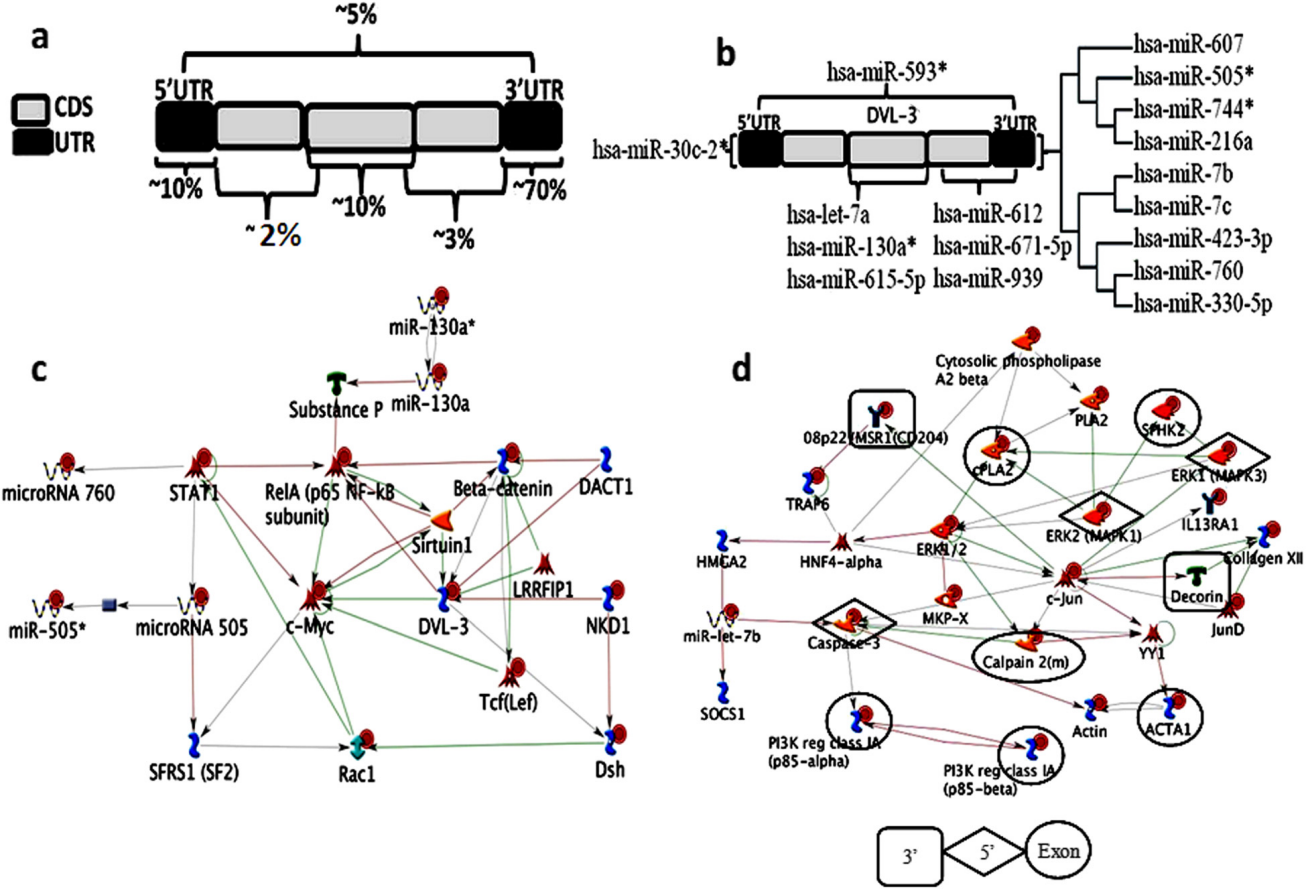
**Schematic representation of miRNA targets deciphered by miRanda and miRNA interaction with Dvl 3 target gene. a**. Represents percentage target hits by miRNAs of different pathway genes at 5'UTR, exons and 3'UTR compiled in oncomiRdbB. **b**. shows an example where several miRNAs are targeting Dvl3 gene at its various positions. **c**. Network interaction of miRNAs in regulating Dvl3 target gene. **d**. Shows miRNA let-7b targeting multiple genes at different positions, 5'UTR, 3'UTR and exons.

We next characterized microRNA target locations within the genes and found that almost 70% of miRNAs target the 3′UTR, 10% of them target the 5′UTR and another 10% were found to target the exons. 5% of miRNAs targeted exons at their 3′ and 5′ UTR, respectively within the gene and the remaining 5% targeted 5′UTR, 3′UTR & exons (Figure 5a). There were many mRNAs which were targeted by multiple miRNAs and among such targets, the Dvl3 gene in the wnt pathway was chosen as an example for further analysis. Interestingly, a number of microRNAs were found to target this gene at its 3′UTR, 5′UTR, exons, or both 3′UTR & 5′UTR or both 3′UTR & exons. The microRNAs targeting the 3′UTR of Dvl3 showed a phylogenetic relationship when their sequences were aligned using ClustalX (Figure 5b). Regulation of Dvl3 by these sets of microRNAs was also deciphered using GeneGo MetaCore (Figure 5c). On the other hand, single miRNAs hitting multiple targets at various genomic locations such as 3′UTR, 5′UTR and exons were also identified. One example, miR-let-7b, had multiple targets at all positions as shown schematically in Figure 5d. Those microRNAs targeting exons and 5′UTR were seen to target key regulators of oncogenic signaling pathways (Additional file 2: Figure S2). The interaction between such microRNAs and their targets in various signaling pathways were delineated using GeneGo MetaCore. The microRNA-target networks and hubs and their therapeutic targets were also deciphered (Figure 3a and Additional file 3: Figure S3a and b).

### Generation of pathway maps using enrichment analysis

Enrichment analysis was performed for the predicted microRNAs and their targets to further support their involvement in breast cancer development in both human and mouse using GeneGo MetaCore. This analysis statistically enriched the miRNAs and their targets with the available dataset in GeneGo MetaCore and generated *p*-values where high negative log *p*-values of microRNAs and their targets reflected significant association with breast oncogenesis. The enrichment analysis also supported our percentage co-operative analysis of different oncogenic pathways in breast cancer development and progression (Figure 3b and c). Similarly, further enrichment analysis for relative expression of these novel miRNAs in different disease conditions in humans showed the highest score for immunoproliferative disorders followed by lymphoproliferative disorders (Figure 3d). The spectrum of miRNAs was found to be crucial in GM-CSF signaling as depicted by the pathway maps generated using MetaCore software (Figure 3e).

### OncomiRdbB database structure

OncomiRdbB is fully designed and developed as a web interface where Perl-CGI is used to connect the Apache web server which works as a back-end to generate dynamically user friendly HTML front end queries, an open-source graphics system that compiles into java code. OncomiRdbB provides easy search options for users to retrieve meaningful information of oncomiRs of two different genomes, their mature sequences, accession no, their respective targets in different oncogenic pathways, location in chromosome and their respective genes. Users can search query by name, accession no, target gene and sequence of miRNAs. It also facilitates downloading of specific microRNAs along with their target details or the complete list of microRNAs and their target information depending on the query. It provides useful interaction data for these microRNAs and their targets in various signaling pathways at the 3′UTR, 5′UTR and exons. Finally this database also provides links to the parental biological databases from which the information is retrieved. OncomiRdbB is hosted on the web and is freely accessible to those who wish to retrieve the information compiled in this database.

## Utility and discussion

MicroRNAs are involved in the regulation of important signaling pathways by controlling the expression of a variety of oncoproteins which are responsible for cancer development and progression [23]. A few existing databases listed these microRNAs and their association with many diseases, including different cancers from diverse organisms as described above. However, none of these specifically describe the class of oncomiRs and their targets for a given cancer. Due to the lack of a properly compiled database, these data remain under utilized. Therefore, we present here a database, OncomiRdbB (OncomiR **d**ata**b**ase for **B**reast cancer), which contains all the mined and compiled information along with ~400 validated oncomiRs of breast cancer. We also report additional novel oncomiRs experimentally identified in human cancer samples, along with their specific target genes. Compared to miRBase, PhenomiR and miR2Disease, OncomiRdbB collates significantly more microRNAs and their targets in both human and mouse. Thus OnmiRdbB provides better compiled information regarding the oncomiRs involved in breast cancer.

MicroRNAs with oncogenic or tumor-suppressive function are capable of modulating several targets in multiple genetic pathways. Mouse models are the predominant mammalian platform chosen to model cancer and as such they represent ideal platform to study the microRNA cancer association in human and to validate targets [24]. With this premise, we performed sequence alignment using ClustalX in order to find any evolutionary relationships which would suggest functional similarity between these miRNAs. Most of the miRNAs of human and mouse formed tight phylogenetic clusters. Comparative cluster analysis additionally confirmed that these miRNAs have common biological functionality and possess similar mechanisms of action in breast cancer. This analysis also interrogated any conserved functional relationships in the development of breast cancer. For example, hsa-miR-145, hsa-miR-151-3p and hsa-miR-30 showed sequence and functional similarity with mmu-miR-145, mmu-miR-151-3p and mmu-miR-30 respectively.

MicroRNA target identification is an important step in determining its specific roles in regulating a cellular process. MicroRNAs are known to regulate mRNAs post-transcriptionally by binding at the 3′ UTR. However, miRNA target prediction in animals is still in its infancy due to limited knowledge about parameters involved in the interaction between miRNAs and their target. Also, the uneven distribution of miRNA binding locations within the target transcript poses a further challenge to the sensitivity and efficiency of computational prediction [25]. Therefore, finding a consistent microRNA target across different miRNA target prediction tools is a challenging task [26]. In this study we used miRanda at different energy levels and PicTar and TargetScan algorithms to minimize the false positive rates during miRNA target prediction. Apart from the complementarity between the mRNA and miRNA, the miRanda algorithm also takes into account the weighted sum of match and mismatch scores for base pairs and gap penalties, allowing one wobbling pair in the seed region. Thus, use of this algorithm takes into consideration different characteristics of the miRNA-mRNA interaction [27]. The miRanda program predicted ‘perfect’ pairing of miRNA-mRNA at lower energy (EL-25) levels, while not ‘so-perfect’ pairing at higher energy levels (EL-15). Thus, miRNA-target prediction at different energy levels will help users to identify putative targets of all miRNAs and also aid validation of specific breast cancer targets in both genomes.

In OncomiRdbB, a total of 711 human and 490 mouse targets are listed which is high compared to the targets listed in other databases. One of the additional features of OncomiRdbB is that these targets are also classified based on various oncogenic signaling pathways which is not yet available in other databases. McCubrey *et al*., [28] have shown that there is interaction among Ras/Raf/MEK/ERK/PI3K/PTEN and Akt pathways during carcinogenesis. Likewise, cooperative interaction of Notch and Ras/MAPK pathway in human breast carcinogenesis and hepatocarcinogenesis was demonstrated by Fan *et al*., [29]. Therefore, the percentage cooperative analysis was carried out to address the role of miRNAs in deregulating multiple oncogenic pathway genes in breast cancer development along with the percentage of target hits by the miRNAs among the total number of targets present in each pathway. Around 30–50 percent target hits in Notch, VEGF, Wnt, Apoptosis, MAPKinase and JAK-STAT pathways indicated a significant intervention by oncomiRs in these pathways during breast cancer development. Although the exact percentage cooperative analysis has not been demonstrated, it is evident that these miRNAs are deregulating various oncogenic pathways. The information generated also led to the identification of candidate microRNAs regulating multiple therapeutic targets to combat breast cancer effectively.

Although the majority of miRNAs have been reported to target the 3′UTR, Zhou *et al*., [30] identified miRNAs targeting coding region and 5′ UTR, but these were less represented and less effective in translation repression. Inhan *et al*., [31] showed that while most of the miRNAs interact with 3′UTR of the target genes with its 5′end, a small proportion of miRNAs binding to 5′UTR use the 3′end for effective interaction. Hence, in order to define the interacting locations of miRNAs within the target genes, 3′UTR, 5′UTR and exon sequences were downloaded and used for target location identification. This analysis indicated that majority (around 70%) lie in the 3′UTR, whilst 10% miRNAs target the 5′UTR, 10% targeted exons, 5% of miRNAs targeted exons at their 3′ and 5′ UTR respectively within the gene and the remaining 5% targeted 5′UTR, 3′UTR & exons. Interaction between miRNAs and their targets was analyzed using the ‘R’ algorithm. Through this statistical analysis program, it was observed that either multiple targets are being controlled by a single miRNA or that several miRNAs target single transcripts. Nevertheless, the former situation was found to be a more common phenomenon than the latter. Dvl3 is one example of a target gene where several miRNAs act at the 3′UTR, 5′UTR and exons. A phylogenetic clustering of the 3′UTR miRNAs indicated a conserved functional relationship amongst them. On the other hand, we observed that a single miRNA, let-7b, hit multiple targets of several oncogenic pathways at their 3′UTR, 5′UTR and exons. It was also observed that targets with location in 5′UTR and exons were the key players of oncogenic pathways.

The interaction of miRNAs and their respective targets was deciphered using the GeneGo MetaCore software suite to identify miRNAs with multiple potential targets for therapeutic intervention in breast cancer. The interactive networks and hubs revealed functional co operativity of several miRNAs and target groups in the various signaling pathways. Engels *et al*., [32] also demonstrated that several miRNAs bind at 3′UTR targets and inhibit translation via co-operative actions [33]. Our analysis also corroborated with this and revealed the possibility of using multiple targets for possible therapeutic intervention. To the best of our knowledge, information on the cooperative interaction of miRNAs and their targets is a unique feature of OncomiRdbB. To support the authenticity of our database as specific to breast cancer, we have performed enrichment analyses of miRNAs and their targets from different oncogenic signaling pathways and provided the degree of relevance of miRNAs and their targets based on *p*-values, where lower *p*-values were assigned higher priority (GeneGo MetaCore software). These *p*-values indicated the probability of a given number of miRNAs and target genes matching with a certain number of miRNAs and target genes responsible for given disease and signaling pathways available in the GeneGo MetaCore suite. The target enrichment analysis supported our hypothesis that there is co-operation among different oncogenic pathways during cancer progression and it also indicated the extent of their potential involvement in cancer progression. This further suggested that our miRNA database for human and mouse could be an acceptable methodology for probing breast cancer miRNAs interrelationships.

We have experimentally validated our database by 2 different array platforms using human breast cancer samples. Approximately 400 miRNAs were valid and significant in one or the other stage/grade. In addition to these, this approach identified 34 novel miRNAs which are not yet reported as breast cancer miRNAs in any of the databases so far. The target identification of these novel miRNAs using miRanda algorithm at 3 energy levels together revealed 162 gene targets. The interaction between the novel miRNAs and their respective targets using MetaCore software delineated the networks and the hubs involved in various oncogenic signaling pathways. Anti-apoptotic molecules such as BCLXW, BCL-Xl were found to be down regulated by the novel miRNAs identified in this study (as predicted by GeneGo pathway), indicating possible roles of these miRNAs in development of cancer and metastatic progression. For example, miR-337-5p, miR-17-1, miR-15a, miR-491-5p, miR-339, miR-337-3p, miR-241, miR-19a were found to modulate oncogenic targets including TGFβ, STATs, c-MYC and SMAD. Taken together, this established a clear mechanistic interaction underlying miRNA deregulation and tumorigenesis.

Our data also indicated potential functional cooperativity of miRNAs and their targets in the development of breast cancer [34]. Hence our finding of cooperation among pathways was supported by the GeneGo MetaCore pathway interaction enrichment analysis. Moreover, we have also deciphered various potential therapeutic targets and their networks. More recently, it has been shown that circulating miRNAs are potential biomarkers for the detection and subsequent staging and grading of breast cancer [35,36]. Unsupervised hierarchical cluster analysis of the miRNA expression between cancerous and normal tissue samples from patients showed major differences in miRNA expression. This study provides a basis for the blood-borne testing of miRNAs as biomarkers for the detection and subsequent staging of breast cancer. Thus, the *in silico* cum experimental validation data regarding the novel miRNA generated using OncomiRdbB could be used for future miRNA-based biomarkers and/or targeted therapeutics.

## Conclusions

OncomiRdbB is designed as a comprehensive user friendly database which lists miRNAs and their respective targets for breast cancer in both the human and mouse. We have experimentally validated ~400 miRNAs in human breast cancer tissues and found 34 miRNAs which are as yet unreported. Users can retrieve information using miRNA name, sequence, accession no., gene name, organism or simply cancer type. OncomiRdbB gives details about the target genes, their chromosome location and position of the miRNA hits within the gene from various oncogenic signaling pathways. This is the first attempt to delineate the complicated interaction network involving different miRNAs and their targets in five oncogenic signaling pathways for breast cancer. OncomiRdbB is a central resource for cancer biologists and clinicians for further experimental validation of these targets and will also help clinicians in the selection of potential candidates for the development of novel clinical biomarkers and ultimately novel therapeutic interventions.

## Availability and requirements

This database is freely available at http://tdb.ccmb.res.in/OncomiRdbB/index.htm. There is no restriction of use for non-academics.

### Ethical clearance

This work has been conducted with the approval of human ethical committees of RCC and CCMB, respectively.

## Competing interests

The authors have declared no financial and non-financial competing interests.

## Authors’ contributions

RK, VKV and AR developed the database under the guidance of LDK, ST and ARC. RAN collected and graded the samples, LDK and RAN designed the experimental validation, LDK and VKV wrote the paper, ARC edited the document, RK, VKV, MG and MMI generated pathway maps in GeneGo MetaCore, AR incorporated all changes in the database as per reviewer’s comments and is responsible for updating and maintaining the database. All authors read and approved the final manuscript.

## Supplementary Material

Additional file 1: Figure S1 A phylogenetic functional relationship between mi.RNAs of human and mouse using ClustalX: Tight clustering showing the phylogenetic relationship of breast cancer miRNAs in human and mouse as depicted by ClustalX. In the cladogram, branches from the same node represent descendents of a similar ancestor or cluster of the same family indicating their origin from a common ancestor. Click here for file

Additional file 2: Figure S2 MiRNA targets interaction in oncogenic pathways. MiRNAs targeting different ongogenic pathways are shown here. Those microRNAs targeting exons and 5′UTR were seen to target key regulators of oncogenic signaling pathways. These miRNAs could be designated as key regulators of Wnt signaling pathways. Click here for file

Additional file 3: Figure S3 Interaction of different signaling pathways and therapeutic targets depicted using metacore 3a: Represents microRNA-target network interaction and their hubs between several oncogenic pathways and their cooperation in breast cancer development. Figure 4b depicts therapeutic targets in different signaling pathways as deciphered by MetaCore software suite. Click here for file
