# Supplementary figures and images for: OncomiRdbB: a comprehensive database of microRNAs and their targets in breast cancer
Source: BMC Bioinformatics. 2014 Jan 15;15:15. doi: 10.1186/1471-2105-15-15 (PMC3926854; doi:10.1186/1471-2105-15-15)

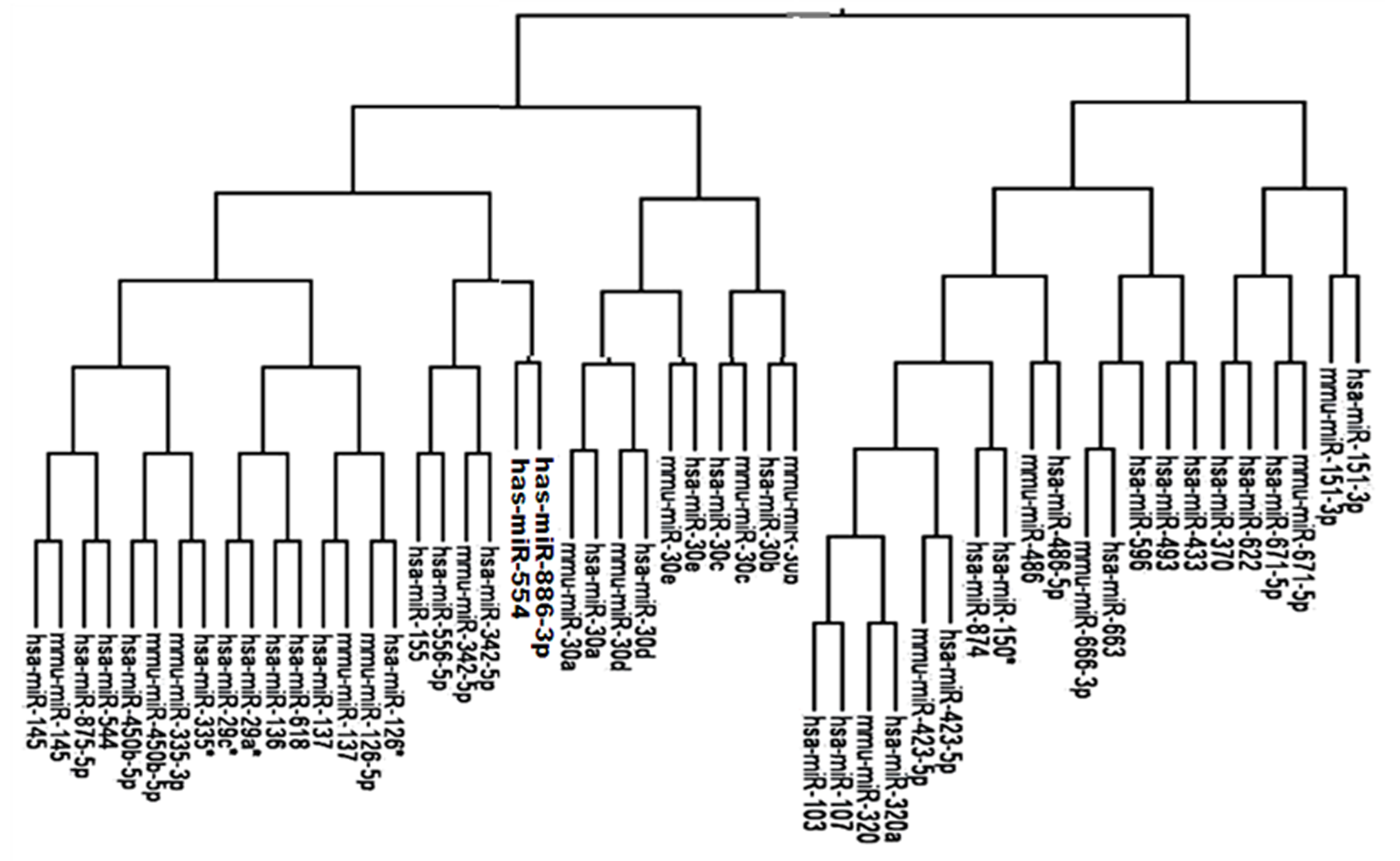

Supplement: Additional file 1: Figure S1 — A phylogenetic functional relationship between mi.RNAs of human and mouse using ClustalX: Tight clustering showing the phylogenetic relationship of breast cancer miRNAs in human and mouse as depicted by ClustalX. In the cladogram, branches from the same node represent descendents of a similar ancestor or cluster of the same family indicating their origin from a common ancestor. [file 1471-2105-15-15-S1.tiff]

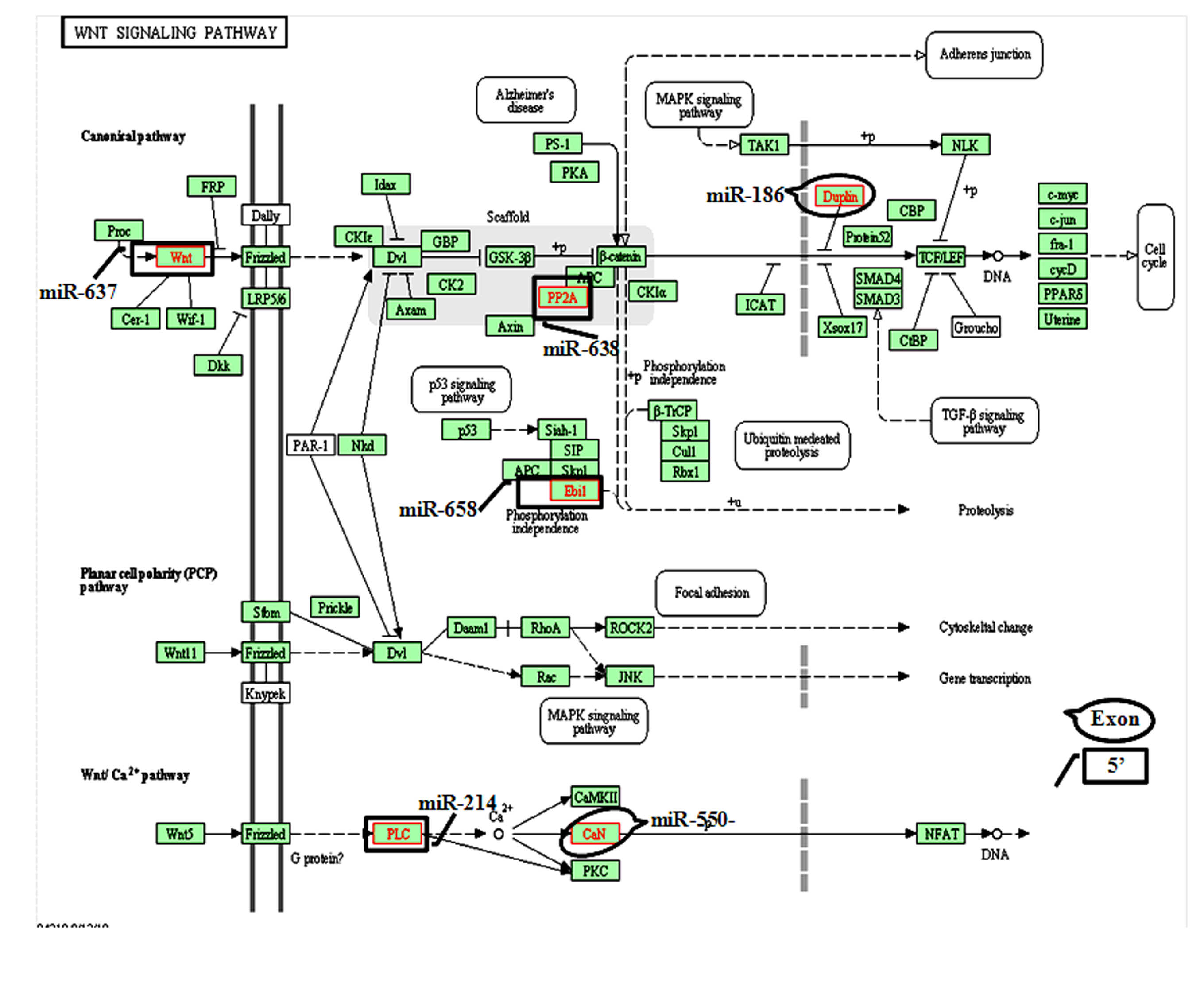

Supplement: Additional file 2: Figure S2 — MiRNA targets interaction in oncogenic pathways. MiRNAs targeting different ongogenic pathways are shown here. Those microRNAs targeting exons and 5′UTR were seen to target key regulators of oncogenic signaling pathways. These miRNAs could be designated as key regulators of Wnt signaling pathways. [file 1471-2105-15-15-S2.tiff]

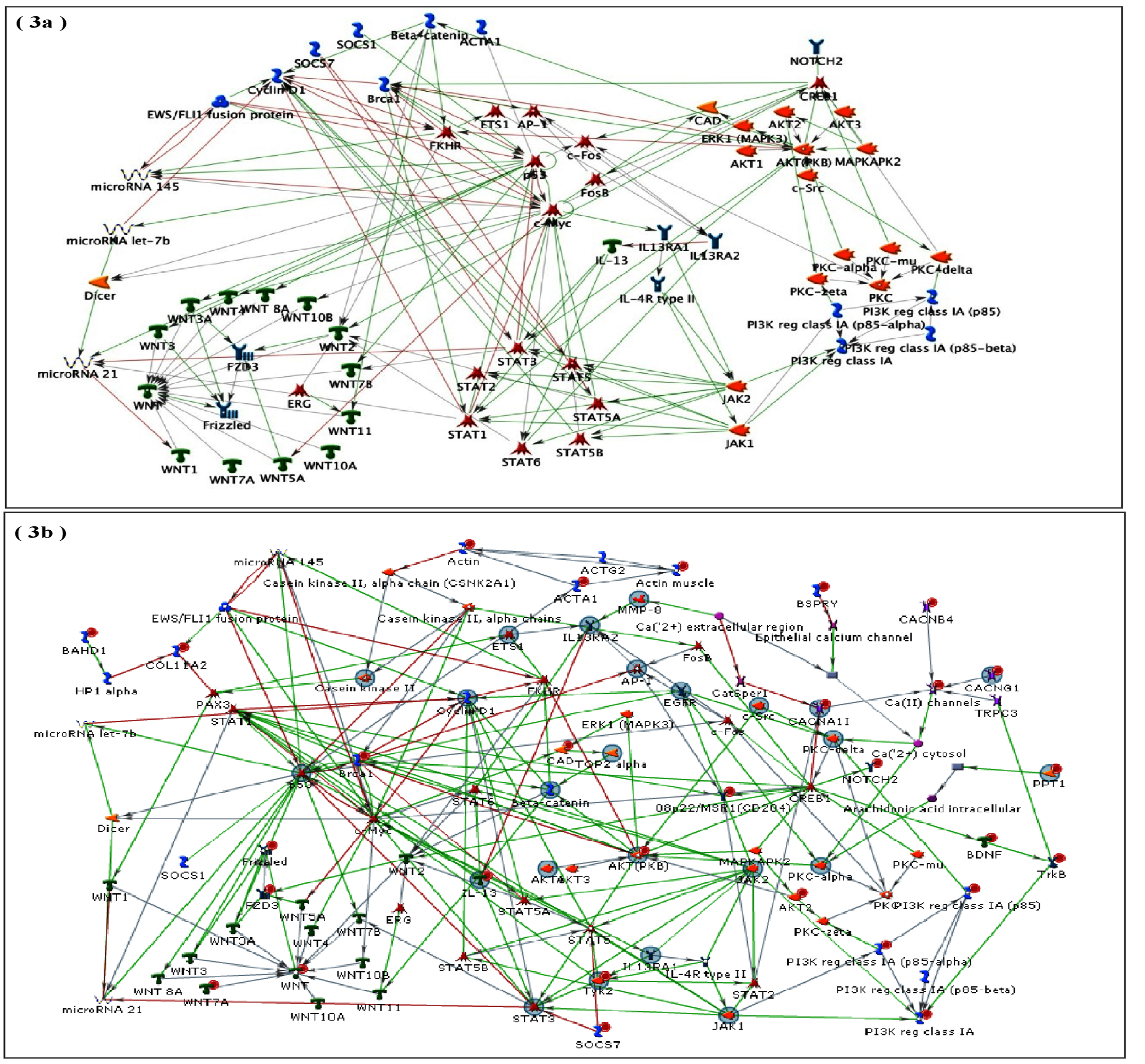

Supplement: Additional file 3: Figure S3 — Interaction of different signaling pathways and therapeutic targets depicted using metacore 3a: Represents microRNA-target network interaction and their hubs between several oncogenic pathways and their cooperation in breast cancer development. Figure 4b depicts therapeutic targets in different signaling pathways as deciphered by MetaCore software suite. [file 1471-2105-15-15-S3.tiff]
